# Vibration acceleration promotes bone formation in rodent models

**DOI:** 10.1371/journal.pone.0172614

**Published:** 2017-03-06

**Authors:** Ryohei Uchida, Ken Nakata, Fuminori Kawano, Yasukazu Yonetani, Issei Ogasawara, Naoya Nakai, Tatsuo Mae, Tomohiko Matsuo, Yuta Tachibana, Hiroyuki Yokoi, Hideki Yoshikawa

**Affiliations:** 1 Department of Orthopedic Surgery, Osaka University Graduate School of Medicine, Osaka, Japan; 2 Department of Sports Medicine, Seifu Hospital, Osaka, Japan; 3 Department of Medicine for Sports and Performing Arts, Osaka University Graduate School of Medicine, Osaka, Japan; University of Notre Dame, UNITED STATES

## Abstract

All living tissues and cells on Earth are subject to gravitational acceleration, but no reports have verified whether acceleration mode influences bone formation and healing. Therefore, this study was to compare the effects of two acceleration modes, vibration and constant (centrifugal) accelerations, on bone formation and healing in the trunk using BMP 2-induced ectopic bone formation (EBF) mouse model and a rib fracture healing (RFH) rat model. Additionally, we tried to verify the difference in mechanism of effect on bone formation by accelerations between these two models. Three groups (low- and high-magnitude vibration and control-VA groups) were evaluated in the vibration acceleration study, and two groups (centrifuge acceleration and control-CA groups) were used in the constant acceleration study. In each model, the intervention was applied for ten minutes per day from three days after surgery for eleven days (EBF model) or nine days (RFH model). All animals were sacrificed the day after the intervention ended. In the EBF model, ectopic bone was evaluated by macroscopic and histological observations, wet weight, radiography and microfocus computed tomography (micro-CT). In the RFH model, whole fracture-repaired ribs were excised with removal of soft tissue, and evaluated radiologically and histologically. Ectopic bones in the low-magnitude group (EBF model) had significantly greater wet weight and were significantly larger (macroscopically and radiographically) than those in the other two groups, whereas the size and wet weight of ectopic bones in the centrifuge acceleration group showed no significant difference compared those in control-CA group. All ectopic bones showed calcified trabeculae and maturated bone marrow. Micro-CT showed that bone volume (BV) in the low-magnitude group of EBF model was significantly higher than those in the other two groups (3.1±1.2mm^3^ v.s. 1.8±1.2mm^3^ in high-magnitude group and 1.3±0.9mm^3^ in control-VA group), but BV in the centrifuge acceleration group had no significant difference compared those in control-CA group. Union rate and BV in the low-magnitude group of RFH model were also significantly higher than those in the other groups (Union rate: 60% v.s. 0% in the high-magnitude group and 10% in the control-VA group, BV: 0.69±0.30mm^3^ v.s. 0.15±0.09mm^3^ in high-magnitude group and 0.22±0.17mm^3^ in control-VA group). BV/TV in the low-magnitude group of RFH model was significantly higher than that in control-VA group (59.4±14.9% v.s. 35.8±13.5%). On the other hand, radiographic union rate (10% in centrifuge acceleration group v.s. 20% in control-CA group) and micro-CT parameters in RFH model were not significantly different between two groups in the constant acceleration studies. Radiographic images of non-union rib fractures showed cartilage at the fracture site and poor new bone formation, whereas union samples showed only new bone. In conclusion, low-magnitude vibration acceleration promoted bone formation at the trunk in both BMP-induced ectopic bone formation and rib fracture healing models. However, the micro-CT parameters were not similar between two models, which suggested that there might be difference in the mechanism of effect by vibration between two models.

## Introduction

Non-union and delayed union are clinical complications of bone fractures that can impair activities of daily living and may require additional surgery. Conservative treatments using physical stimuli have been developed. Low-intensity pulsed ultrasound (LIPUS) is one of the useful and representative treatment apparatus to promote fracture healing by its mechanical stimulus, micromotion, at fracture site [1]. In addition, vibration acceleration by whole body vibration (WBV) has positive effects on bone healing and density due to its mechanical stimulus similar to LIPUS, though WBV has been primarily applied to muscle strength exercise and stretching. LIPUS is usually used for small areas of relatively superficial bone layers in the upper or lower extremities, whereas WBV can also be applied to deep bone layers in the trunk.

*In vivo* studies have shown that vibration acceleration can promote bone formation in young adults and decrease bone loss in elderly individuals and postmenopausal women as well as in both small and large animals [2–17]. These effects were predominantly reported in limbs and especially low-magnitude, high-frequency (LMHF) vibration is more effective for increasing bone density and bone union compared with high-magnitude or low-frequency vibration [2–14, 17]. However, there are some acceleration modes including vibration or centrifuge acceleration. Centrifuge acceleration also has effects on bone formation such as increase of tibial cancellous bone and reduction of femoral cortical bone in rats [18, 19]. Both direction and amplitude change regularly in vibration acceleration, whereas these are constant in gravity or centrifuge acceleration, classified as constant acceleration. There have been no reports establishing whether the acceleration mode influences the effect of acceleration on bone formation and healing. Therefore, the purpose of this study was to compare the effect of acceleration on bone formation in the trunk using ectopic bone formation in a dorsal subfascial pocket or a rib fracture model between two modes of acceleration: vibration acceleration mode and constant acceleration mode. Constant acceleration mode included centrifuge acceleration as well as gravitational acceleration. In this study, gravitational acceleration was only baseline and we compared the effect on bone formation between vibration and constant acceleration modes with centrifuge acceleration. In addition to comparison of acceleration mode, we tried to verify the difference in mechanism of effect on bone formation by accelerations between ectopic bone formation (in dorsal subfascial pocket) model and a rib fracture model. BMP-induced ectopic bone formation model was one of highly-reproducible model for investigation of bone formation and easy to apply intervention, whereas the condition in case of natural fracture can be reproduced in rib fracture healing model with bone marrow cells as well as cytokine including BMP at fracture site.

Our hypothesis was that vibration acceleration has a positive effect on bone formation and healing, because the direction and amplitude changes of acceleration are important factors to generate the mechanical stimulus that promote bone formation. However, the effects of accelerations are not same between two models leading to the different results in measured parameters.

## Materials and methods

All experimental procedures were conducted in accordance with the Guide for the Care and Use of Laboratory Animals of the Physiological Society of Japan. The study was also approved by the Animal Use Committee at the Osaka University Graduate School of Medicine (approval ID: 22–071).

This study evaluated the effects of acceleration modes on bone formation and fracture healing using two *in vivo* experimental rodent models: BMP-induced ectopic bone formation (EBF) and rib fracture healing (RFH) models [20–23]. Two acceleration modes, vibration and constant (centrifugal) acceleration, were tested in each model. Testing of the vibration acceleration mode included three groups (low-magnitude, high-magnitude, and control-VA groups), whereas testing of constant acceleration mode included two groups (centrifuge acceleration and control-CA groups). A total of five different acceleration conditions were evaluated in both EBF and RFH models.

### BMP-induced Ectopic Bone Formation (EBF) model

As described previously [22], under anesthesia with an intraperitoneal injection of pentobarbital sodium (5 mg/100 g body weight), the back was sterilized and a longitudinal incision was made over the thoracic spine. While the superficial back muscles were retracted laterally, BMP/atelocollagen pellets containing 3 μg rhBMP-2 (kindly provided by Osteopharma Inc., Osaka, Japan) and 20 mg collagen absorbable hemostat, INSTAT^®^ (Ethicon, Inc., Somerville, New Jersey, USA; discontinued product in Japan) were implanted into a dorsal subfascial pocket of 40 five-week-old male ICR mice (Nihon CREA, Tokyo, Japan). The skin was sutured and the animals were then maintained in cages. The 40 mice were divided randomly into five groups. After operation, we observed feeding quantity and body weight and disinfected the wounds at least once a day. A significant change body weight was not found in mice postoperatively compared to those preoperatively. Each four mice were housed in a cage (20 x 10 cm and 10 cm height). A commercial solid diet (CE-2, Nihon CLEA, Tokyo, Japan) and water were supplied ad libitum. Temperature and humidity in the animal room with 12:12 hr light:dark cycle were maintained at 20~24°C and 40~60%, respectively.

### Rib Fracture Healing (RFH) model

The rib fracture healing model was generated in 25 four-week-old female Wistar rats (Nihon CREA, Tokyo, Japan) using the method previously described [23]. Each rat was anesthetized with ethylether and intraperitoneal injection of pentobarbital sodium (5 mg/100 g body weight). The back was sterilized, and a longitudinal incision was made over the thoracic spine. The superficial back muscles were retracted laterally, exposing the dorsal aspect of the ribs. Then eighth ribs on each side were cut vertically with scissors to the shaft at the lateral margin of the paravertebral muscle. The skin was sutured and the animals were then maintained in cages. Similar to the EBF model, the 25 rats were divided randomly in five groups. After operation, we observed and cared similarly to mice in EBF model. A significant change body weight was not found in rats postoperatively compared to those preoperatively. Each five rats were housed in a cage (28 x 45 cm and 20 cm height). A commercial solid diet (CE-2, Nihon CLEA, Tokyo, Japan) and water were supplied ad libitum. Temperature and humidity in the animal room with 12:12 hr light:dark cycle were maintained at 20~24°C and 40~60%, respectively.

### Acceleration settings

**Fig 1:**
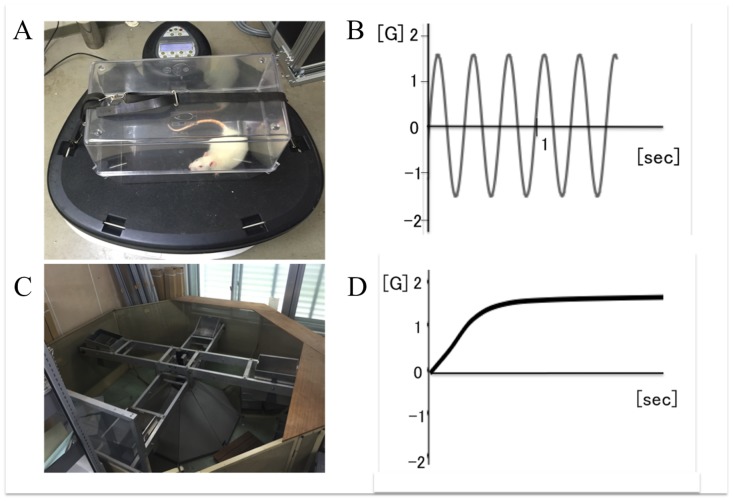
A Power Plate^®^ Pro5HP^™^ (A) and the change (B) of the vibration acceleration intervention and an animal centrifuge (C) and the change (D) of constant centrifugal acceleration intervention.

In both models, control group animals were placed close to the acceleration apparatus to minimize the influence of noise and vibration during acceleration between the experimental and control groups. There were some differences in the stress for rodents caused by noise and indirect vibration from apparatuses (Power Plate^™^ and animal centrifuge) between two acceleration models. Therefore, we set total two different control groups, one in each acceleration model. In vibration acceleration groups, animals in low- (2.5-mm amplitude, 30 Hz) and high- (5-mm amplitude, 30 Hz) magnitude groups were put in a cage on a Power Plate^®^ Pro5HP^™^ (Performance Health Systems, LLC, available through Protea Japan Co. Inc., Tokyo, Japan). Actual acceleration in each group was measured using an accelerometer (Logitec Inc., Chiyoda, Tokyo, Japan) in the cage (Fig 1A). When gravitational acceleration (1G) was set as the baseline and the direction of gravity was considered as positive, the acceleration in low- and high-magnitude groups periodically changed from -1.5G to 1.5G or -3G to 3G, respectively (Fig 1B). In the centrifuge acceleration group, animals were loaded at 1.5G by using an animal centrifuge with four arms (1.22 m arm length) and a swing speed of 33 rpm excluding gravitational acceleration (Fig 1C and 1D). In summary, for both EBF and RFH models, five groups in terms of type of acceleration (low-magnitude vibration, high-magnitude vibration, centrifuge acceleration and two controls groups (control-VA and control-CA groups).–one for each model) were set.

These accelerations were applied for ten minutes per day beginning three days after surgery. The intervention continued for eleven days in the EBF model and nine days in the RFH model.

### Evaluations

All samplings were performed after cardiac excision under anesthesia with intraperitoneal injection of sodium pentobarbital (5 mg/100 g body weight). In the EBF model, ectopic bone was dissected from the dorsal subfascial pocket and the surrounding soft tissue was carefully removed. Each ectopic bone underwent macroscopic observation, wet weight measurement, radiological assessment by radiography and microfocus computed tomography (micro-CT), and histological evaluation. In the RFH model, both whole eighth ribs were cut from the vertebra and the surrounding soft tissue was excised. Radiological and histological evaluations were performed on the rib fracture sites. Fractured ribs were examined radiologically in two dimensions with a soft X ray apparatus (30 kV. 2 mA, 60 sec of exposure) after sacrifice and judged to be healed or not as described previously [23]. The fracture was judged to be healed when continuity of the cortex was observed at the fracture site in both two dimensions, and not to be healed when an intervening radiolucent zone was observed even in at least one dimension.

### Micro-CT analysis

**Fig 2:**
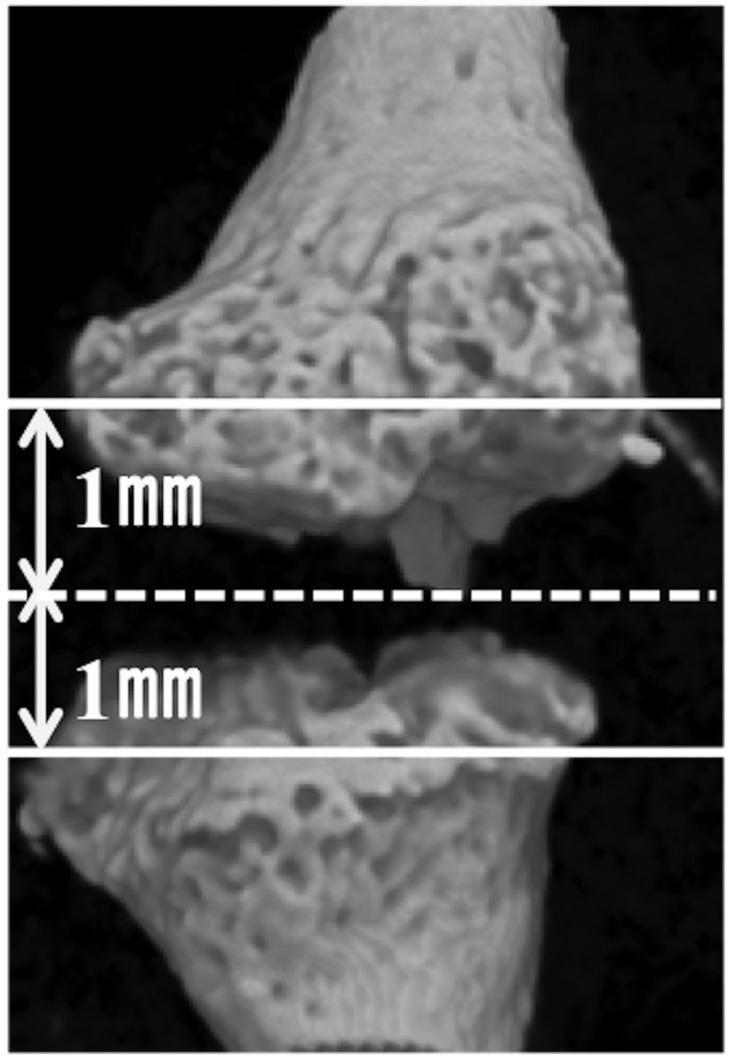
Region of interest in rib fracture for evaluation by micro-CT. ROI was the space between 1-mm proximal and distal planes (white lines) from the center of the bone defect (dotted white line); these planes were perpendicular to the bone axis.

Formation of new bone in each BMP/atelocollagen pellet or fracture site was evaluated using a micro-CT system (SMX-100CT-SV; Shimadzu, Kyoto, Japan). Each sample was scanned at 25-μm intervals at 50 kV and 200 μA. With an angular step of 0.3°over an angular range of 180°, 600 radiographic projections were acquired. The spatial resolution was 24μm. Next, 3-D images were reconstructed with a threshold of 1,000 Hounsfield units using TRI3D-BON software (Ratoc System Engineering, Tokyo, Japan). Region of interest (ROI) in EBF model was whole ectopic bone. In RFH model, ROI was the space between 1-mm proximal and distal planes from the center of the bone defect; these planes were perpendicular to the bone axis (Fig 2). Evaluation items of new bone formation were bone volume (BV), bone volume per total volume (BV/TV), bone mineral density (BMD), trabecular thickness (Tb.Th), trabecular number (Tb. N), trabecular separation (Tb.Sp) [24].

### Histological analysis

Samples were decalcified with ethylenediaminetetraacetic acid (pH 7.4) after 10% neutral formalin fixation. After decalcification, the samples were dehydrated in a graded ethanol series, cut along the coronal plane at the center of ectopic bones or at the midline of the defect in fracture site, and embedded in paraffin. Sections (3 μm thickness) were each mounted on individual slides, and stained with hematoxylin and eosin (H&E) or safranin-O for observation under a light and polarized light microscope (Eclipse 90i; Nikon, Tokyo, Japan).

### Statistical analysis

All data are expressed as mean ± standard deviation (SD). The Mann-Whitney U test was used for statistical analysis of differences between groups. A statistical difference between experimental groups was regarded as significant when the p-value was <0.05.

## Results

Body weights of animals before surgery and at sacrifice did not show any significant differences between groups in each model (Tables 1 and 2).

**Table 1:** Animal body weight in ectopic bone formation model.

	Vibration acceleration			Constant acceleration	
	Control-VA (n = 8)	Low (n = 8)	High (n = 8)	Control-CA (n = 8)	Centerfuge (n = 8)
Body weight before surgery (mg)	35.0±1.5	35.4.±0.8	33.6.±0.8	34.6±1.2	34.1±0.9
Body weight at sacrifice (mg)	37.2±1.6	38.8±1.2	40.8±1.0	38.2±0.7	39.5±1.1

Control-VA: Control group of vibration acceleration groups, Low: Low-magnitude group, High: High-magnitude group, Control-CA: Control group of constant acceleration groups, Centrifuge: Centrifuge group

**Table 2:** Animal body weight in rib fracture healing model.

	Vibration acceleration			Constant acceleration	
	Control-VA (n = 5)	Low (n = 5)	High (n = 5)	Control-CA (n = 5)	Centrifuge (n = 5)
Body weight before surgery (mg)	83.6±4.2	84.0±4.0	84.8±3.6	84.2±4.1	84.6±4.4
Body weight at sacrifice (mg)	140.3±6.7	143.5±4.7	142.1±8.4	141.8±6.2	142.2±5.9

Control-VA: Control group of vibration acceleration groups, Low: Low-magnitude group, High: High-magnitude group, Control-CA: Control group of constant acceleration groups, Centrifuge: Centrifuge group

### EBF model

**Fig 3:**
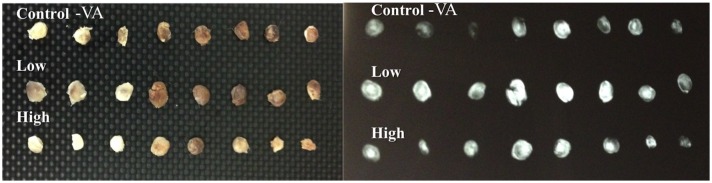
Macroscopic (left panel) and radiographic (right panel) aspects of ectopic bones among three groups (Control-VA: Control group of vibration acceleration groups, Low: Low-magnitude group, High: High-magnitude group).

**Fig 4:**
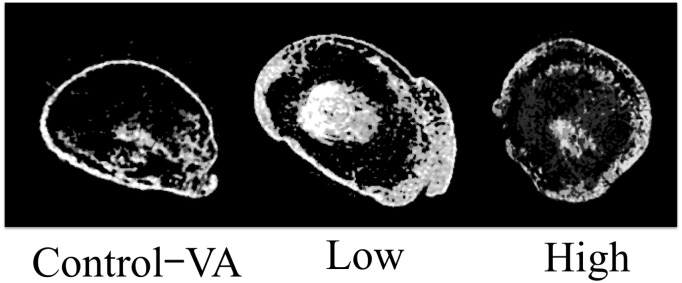
CT images of ectopic bones in three groups of ectopic bone formation model. New bone formations were found in all three images with difference in bone quantity among three groups. Control-VA: Control group of vibration acceleration groups, Low: Low-magnitude group, High: High-magnitude group.

In vibration acceleration groups, ectopic bones in the low-magnitude group were macroscopically and radiographically larger than those in the high-magnitude and control-VA groups (Fig 3). The mean wet weight of ectopic bones in the low-magnitude group was significantly heavier than those in the other groups (59.1 ± 16.8 mg in the low-magnitude group vs. 30.1 ± 11.8 mg in the high-magnitude group and 31.7 ± 11.1 mg in the control-VA group). Micro-CT showed that BV in the low-magnitude group was larger than those in the control-VA and high-magnitude groups. Moreover, BV in the low-magnitude group was also larger than those in the control-CA and centrifuge groups. (Table 3, Fig 4, S1 Fig).

**Table 3:** Micro-CT analysis of ectopic bone among vibration and constant acceleration groups.

Vibration acceleration				Constant acceleration	
	Control-VA (n = 8)	Low (n = 8)	High (n = 8)	Control-CA (n = 10)	Centrifuge (n = 10)
BV [mm^3^]	1.3±0.9	3.1±1.2 ^a^^,^ ^b^	1.8±1.2	1.5±1.2	1.4±1.0
BV/TV [%]	36.5±11.4	33.2±10.7	45.9±14.2	35.5±10.3	33.7±11.1
BMD [mg/cm^3^]	233.6±18.7	240.0±74.0	235.8±10.0	253.6±19.5	262.0±80.0
Tb.Th [μm]	46.7±8.9	52.0±8.0	53.9±9.2	48.1±7.2	47.9±9.1
Tb.N [1/mm]	7.9±2.4	6.6±2.5	8.6±2.6	6.8±4.4	7.9±2.6
Tb.Sp [μm]	95.5±53.7	126.8±72.1	74.8±43.2	105.0±55.8	118.8±64.3

Control-VA: Control group of vibration acceleration groups, Low: Low-magnitude group, High: High-magnitude group, Control-CA: Control group of constant acceleration groups, Centrifuge: Centrifuge group

^a^ Values in the low-magnitude group were significantly higher than those in the control-VA, and high-magnitude group (vibration acceleration groups).

^b^ Values in the low-magnitude group were significantly higher than those in the control-CA and centrifuge group (constant acceleration groups).

**Fig 5:**
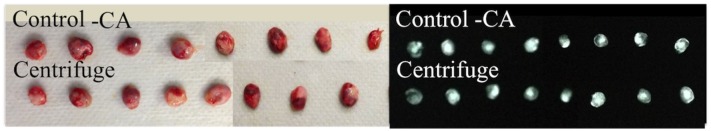
Macroscopic (left panel) and radiographic (right panel) aspects of ectopic bones between two groups (Control-CA: Control group of constant acceleration groups, Centrifuge: Centrifuge group).

In constant acceleration groups, there were no parameters with significant difference in the size of ectopic bone between the control-CA and centrifuge acceleration groups macroscopically or radiographically (Fig 5). Moreover, the mean wet weights of ectopic bones in the two groups have no significant difference (31.6± 7.5 mg in the centrifuge acceleration group vs. 34.0 ± 6.2 mg in the control-CA group). None of the micro-CT parameters were significantly different among the other groups including vibration acceleration groups (Table 3, S1 Fig).

**Fig 6:**
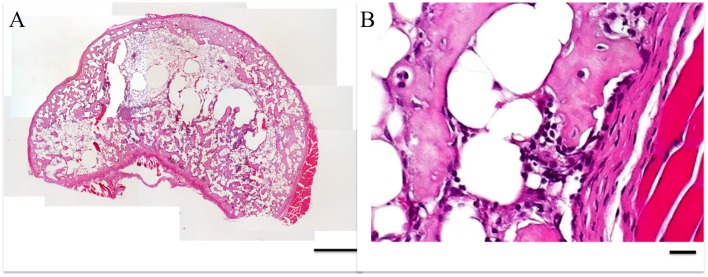
Microscopic images of ectopic bone cross-sections stained with hematoxylin and eosin (H&E) at (A) 40X, scale bar = 500 μm and (B) 400X, scale bar = 100 μm.

Histologically, all ectopic bones in all five groups were similar to those in previous reports. The calcified trabeculae and maturated bone marrow were visible (Fig 6).

### RFH model

In vibration acceleration groups, the union rate of rib fracture in the low-magnitude group was higher than that in the other two groups (60% vs. 0% in the high-magnitude group and 10% in the control-VA group) (S2 Fig). Results of micro-CT showed that BV in the low-magnitude group was larger than those in the other two groups and BV/TV in the low-magnitude group was higher than that in the control-VA group. BV in the low-magnitude group was also larger than those in the control-CA and centrifuge groups similar to EBF model (Table 4).

**Table 4:** Micro-CT analysis of rib fracture sites among vibration acceleration groups and constant acceleration groups.

Vibration acceleration				Constant acceleration	
	Control-VA (n = 8)	Low (n = 8)	High (n = 8)	Control-CA (n = 10)	Centrifuge (n = 10)
BV [mm^3^]	0.22±0.17	0.69±0.30 ^a^^,^ ^b^	0.15±0.09	0.25±0.33	0.26±0.13
BV/TV[%]	35.8±13.5	59.4±14.9 ^c^	39.1±13.9	37.2±15.2	37.8±13.8
Tb.Th [μm]	44.8±10.9	55.0±8.6	40.0±7.7	46.5±9.30	43.3±15.5
Tb.N[1/mm]	7.6±1.0	11.0±3.3	9.8±3.6	9.2±3.1	9.7±2.5
Tb.Sp [μm]	86.3±27.6	44.3±27.9	79.7±54.9	72.1±20.3	68.8±25.4

Control-VA: Control group of vibration acceleration groups, Low: Low-magnitude group, High: High-magnitude group, Control-CA: Control group of constant acceleration groups, Centrifuge: Centrifuge group

^a^ Values in the low-magnitude group were significantly higher than those in the control-VA, and high-magnitude group (vibration acceleration groups).

^b^ Values in the low-magnitude group were significantly higher than those in the control-CA and centrifuge group (constant acceleration groups).

^c^ Values in the low-magnitude group were significantly higher than those in the control-VA group.

In constant acceleration groups, the union rates of rib fracture between the centrifuge acceleration and control-CA groups have no significant differences (10% in the centrifuge acceleration group vs. 20% in the control-CA group) (S2 Fig). Similar to the EBF model, none of the micro-CT parameters were significantly different among the other groups including vibration acceleration groups (Table 4).

**Fig 7:**
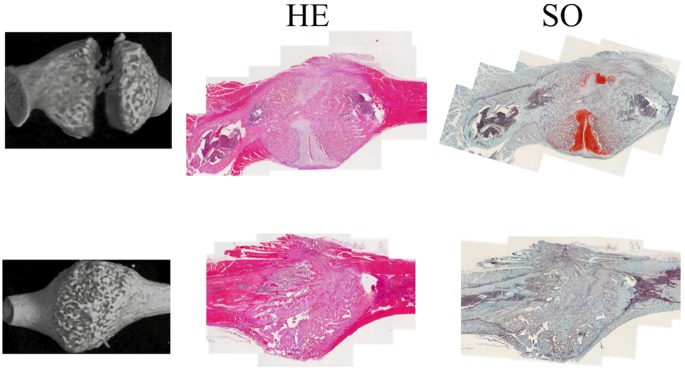
Microscopic images of fracture callus stained with hematoxylin and eosin (H&E) and safranin-O. Representative images of non-union ribs are in the upper row and those of union in the lower row: micro-CT images (left column), histological images stained with H&E (middle column) and histological images stained with safranin-O (right column). Magnification = 40X, scale bar = 1 mm.

Histologically, in radiographic non-union samples, cartilage filled the defect at the fracture site and new bone formation was poor, whereas only new bone filled in the defect in radiographic union samples. The osteoid was not observed in both non-union and union samples and no clear differences in cells associated with fracture healing such as osteoblast and osteoclasts in new bone formation region were found between both samples. (Fig 7).

## Discussion

The positive effects of acceleration by WBV training on bone healing or density have recently been established. Vibration acceleration can also promote *in vivo* bone formation in humans and animals [2–17]. All living tissues and cells on earth are subject to various modes of acceleration, including gravitational acceleration. However, there have been no reports investigating the influence of acceleration mode, such as vibration acceleration or constant acceleration, on bone formation and healing. This study showed a positive effect of vibration acceleration on bone healing and formation, whereas constant acceleration by gravity and/or centrifuge force did not have significant effect. To our knowledge, this study is the first to compare the effects of two different modes of acceleration stimulation, vibration acceleration and constant acceleration, on bone healing and formation. Moreover, our results confirmed a difference between low- and high-magnitude vibration in promoting bone healing and formation. This study also showed that WBV had a positive effect on bone formation even in deep layers of the trunk, in contrast to LIPUS for bone healing in superficial layers in the extremities.

A previous study investigating the mechanism of vibration acceleration showed that controlled dynamic loading using varying frequencies (1 to 10 Hz) produced perturbations of the intermedullary pressure in adult female rats [20]. Consequently, fluid flow increases in response to loading, and shear stress on cell membranes caused by fluid flow stimulates bone cells in culture [25, 26]. Extracellular fluid forces can be converted into cellular responses via several different mechanisms [27]. Vibration stimuli have been proposed to provide mechanical loading adequate to increase fluid flow in bone and facilitate mechanotransduction [26, 27]. However, constant acceleration by centrifuge cannot generate adequate stress and fluid flow for bone healing or bone formation. This highlights the importance of applying vibration acceleration with periodic changes in direction and amplitude for bone healing and formation. Consequently, to know the precise mechanisms, further studies need, because this study showed only the positive effect of WBV on bone healing and formation in small animals.

Low-magnitude vibration acceleration promoted bone formation in both EBF and RFH models; however, micro-CT results differed slightly between models. In the EBF model, BV was significantly higher in the low-magnitude vibration group than those in the high-magnitude group and control-VA, while BV/TV remained unchanged. This suggests that the vibration acceleration influenced the quantity of BMP-induced bone matrix formation, possibly due to the promotion of cell migration, proliferation and differentiation of newly produced bone. On the other hand, in the RFH model, BV and BV/TV in the low-magnitude vibration group were significantly higher than those in the other one or two groups. These results suggest that the newly produced bone in RFH model has smaller pores like cortical bone than that in EBF model, as the rib is healing and returning to cortical bone-like structure. The vibration acceleration may also have some influence on the bone marrow cells associated with the differentiation of newly bone at the fracture site, resulting in the higher BV/TV. BMP-induced ectopic bone formation model was one of highly-reproducible model for investigation of bone formation and easy to apply intervention, whereas the condition in case of natural fracture can be reproduced in rib fracture healing model with bone marrow cells as well as cytokine including BMP at fracture site. If the evaluations of two acceleration modes between two models would not be performed, we had not known these results associated with clues to the mechanism of the effect of vibration acceleration on bone formation as well as its effect. Therefore, we adopted two acceleration modes for two models, EBF and RFH models. However, to verify the precise mechanism in detail, further investigations are required.

Previous animal experiments showed that LMHF acceleration by WBV promoted fracture healing and increased bone mineral density [2, 3, 6–8, 9, 10]. Most animal studies reporting positive effects of WBV on bone characteristics employed magnitudes less than 1 G [2, 3, 6–9]. However, some reports were showed the positive effect of magnitude more than 1G on bone formation and bone density. Moreover, Wegner et al. [17] described that the purported anabolic effect of WBV on bone homeostasis may depend on location and the parameter of interest. In this study, the magnitude in the low-magnitude group was 1.5 G, which was more than the previously recommended magnitude of 1 G. However, the target organs in this study were in a dorsal subfascial pocket or the ribs in the trunk whereas most target organs in the previous studies were in the lower extremities or vertebral body so that vibrations could be directly transmitted through the foot. Vibration to the targets in this study might be decreased due to intervenient soft tissue, joint angle and posture. Therefore, 1.5 G using the power plate showed a positive effect on the ectopic bone and fractured ribs. Surprisingly, the duration of vibration for promoting bone formation was only ten minutes per day. The effect of vibration acceleration might continue not only during vibration but also for a certain period of time after the end of vibration. Some chemical or other signals are thought to continue for some period after vibration. Vibration acceleration may increase cell recruitment to the fracture site and trigger some cascades.

We acknowledge that this study has some limitations. First, the vibration magnitude and frequency were limited since our study used a power plate that is a vibration exercise machine for humans. Therefore, we have to modulate vibration frequency and magnitude to determine the optimal conditions for bone healing and formation. Second, the number of rodents in this study was small and thus if we concluded no significant difference from our results, it may be necessary to have the data in large number rodents. However, some significant differences were found in low-magnitude vibration group. Therefore, we concluded that low-magnitude vibration acceleration promoted bone formation and healing. Finally, though this study showed only the positive effect of WBV on bone healing and formation in small animals, the precise mechanisms of the effects are unknown. Regarding histological analysis, further evaluation including of analysis using immunostaining should be performed, but only simple findings was described in this study. In the near future, we hope to investigate the mechanism of vibration acceleration on the bone using an *in vivo* time-course analysis including histological evaluation as well as an *in vitro* study.

## Conclusion

Low-magnitude vibration acceleration promoted bone formation at the trunk in both BMP-induced ectopic bone formation and rib fracture healing models. However, the micro-CT parameters were not similar between two models, which suggested that there might be difference in the mechanism of effect by vibration between two models.

## Supporting information

S1 Fig CT images (MPR slice) of all ectopic bone divided 5 groups. Control (vibration), low-magnitude and high-magnitude acceleration groups in the vibration acceleration study, and control (constant) and centrifuge acceleration groups in the constant acceleration study. Low: low-magnitude acceleration group, High: high-magnitude acceleration group, Centrifuge: centrifugal acceleration group.
(TIFF)

S2 Fig Radiographic images of all fractured-ribs in divided 5 groups. Control (vibration), low-magnitude and high-magnitude acceleration groups in the vibration acceleration study, and control (constant) and centrifuge acceleration groups in the constant acceleration study. Low: low-magnitude acceleration group, High: high-magnitude acceleration group, Centrifuge: centrifugal acceleration group. Union: union ribs (left column), Non-union: non-union ribs (right column). (No union ribs in high-magnitude vibration acceleration group).
(TIFF)
