# Supplementary figures and images for: Vibration acceleration promotes bone formation in rodent models
Source: PLoS One. 2017 Mar 6;12(3):e0172614. doi: 10.1371/journal.pone.0172614 (PMC5338772; doi:10.1371/journal.pone.0172614)

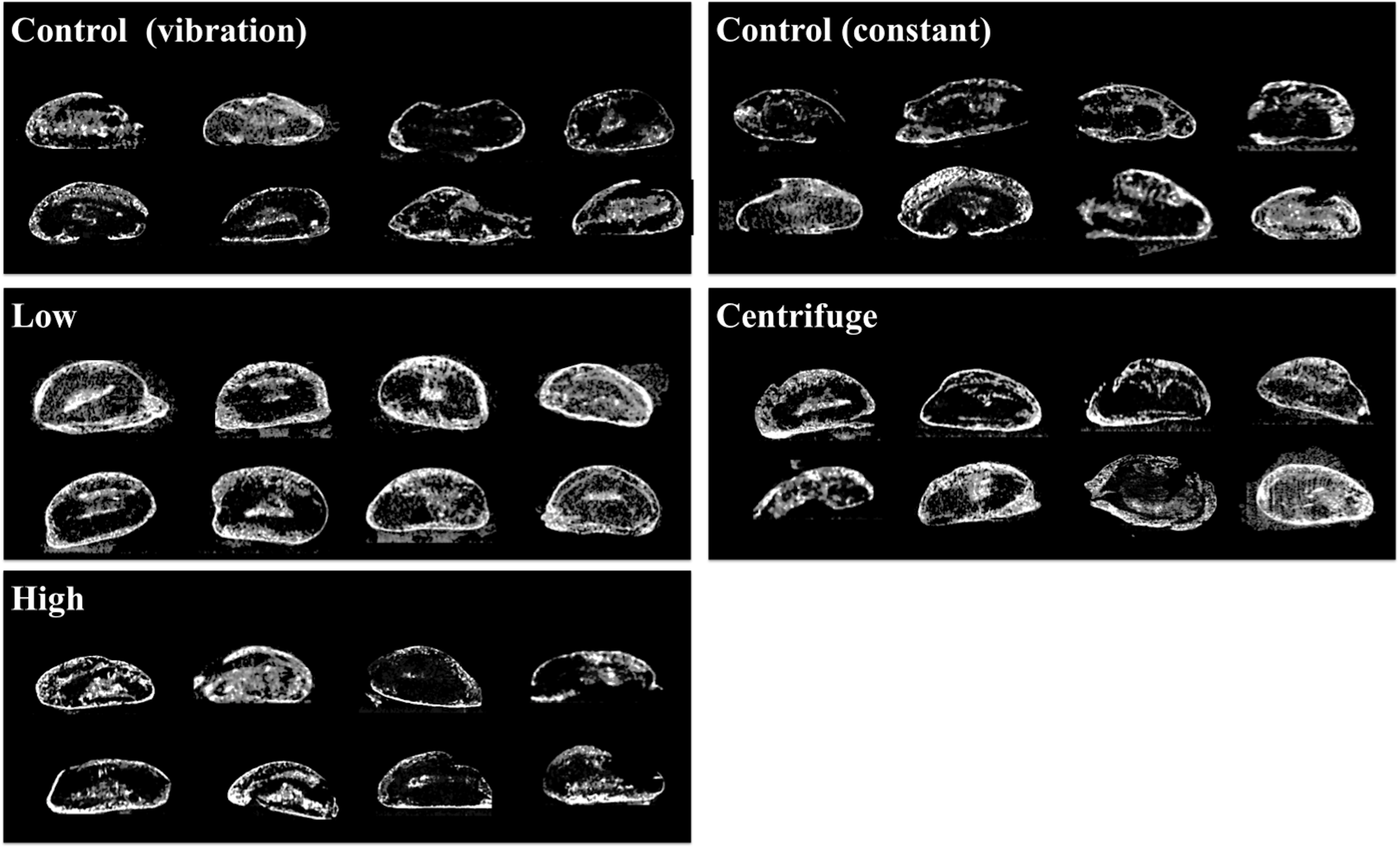

Supplement: S1 Fig — Control (vibration), low-magnitude and high-magnitude acceleration groups in the vibration acceleration study, and control (constant) and centrifuge acceleration groups in the constant acceleration study. Low: low-magnitude acceleration group, High: high-magnitude acceleration group, Centrifuge: centrifugal acceleration group. (TIFF) [file pone.0172614.s001.tiff]

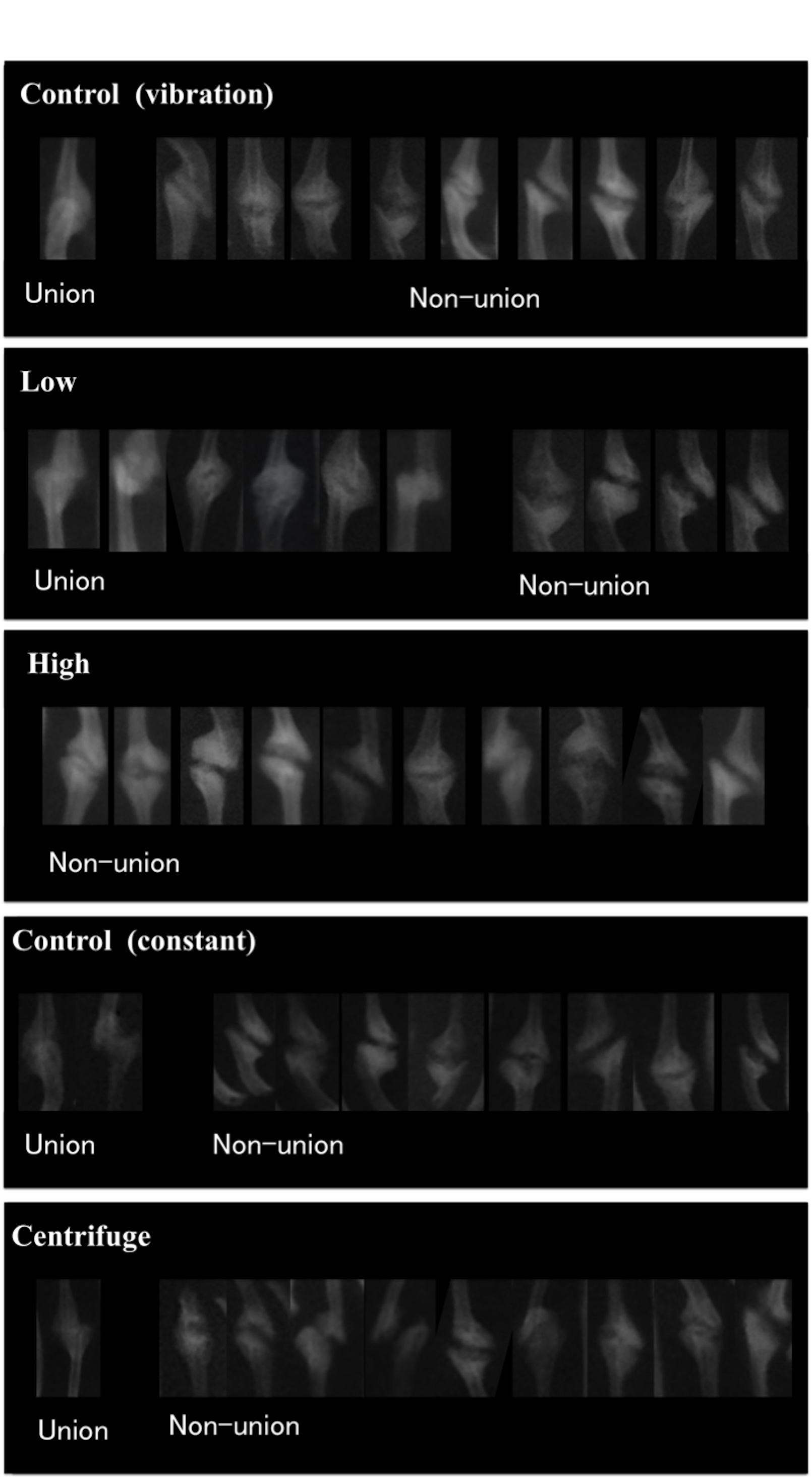

Supplement: S2 Fig — Control (vibration), low-magnitude and high-magnitude acceleration groups in the vibration acceleration study, and control (constant) and centrifuge acceleration groups in the constant acceleration study. Low: low-magnitude acceleration group, High: high-magnitude acceleration group, Centrifuge: centrifugal acceleration group. Union: union ribs (left column), Non-union: non-union ribs (right column). (No union ribs in high-magnitude vibration acceleration group). (TIFF) [file pone.0172614.s002.tiff]
